# 2394 Sodium-glucose transporter 2 is a novel diagnostic and therapeutic target for early-stage lung adenocarcinoma

**DOI:** 10.1017/cts.2018.122

**Published:** 2018-11-21

**Authors:** Claudio Scafoglio, Gihad Abdelhady, Jie Liu, Jane Yanagawa, Dean Wallace, Jorge Barrio, Steven Dubinett, David Shackelford

## Abstract

OBJECTIVES/SPECIFIC AIMS: Lung cancer claims 160,000 lives in the United States every year, and lung adenocarcinoma (LADC) is the most frequent type. Early diagnosis is crucial. Computed tomography (CT) is very sensitive in identifying early-stage lung nodules, but has low specificity. Increased glucose uptake is a hallmark of cancer measurable in vivo by fluorodeoxyglucose (FDG) positron-emission tomography (PET). FDG PET is widely used for cancer staging but has low sensitivity in the diagnosis of solitary lung nodules. We have previously identified an alternative glucose transporter, SGLT2, expressed in different types of cancer but not detected by FDG PET. SGLT2 activity can be measured in vivo with the PET tracer methyl-4-fluorodeoxyglucose (Me4FDG). The objective of this study was to test the hypothesis that SGLT2 is a novel diagnostic and therapeutic target in FDG-negative, early stage LADC. METHODS/STUDY POPULATION: To study glucose transporter expression in LADC, we performed immunohistochemistry with SGLT2- and GLUT1-specific antibodies in human lung pre-malignant lesions and LADC samples. To verify the possibility of detecting SGLT2 activity in vivo, we performed microPET imaging with the SGLT-specific tracer Me4FDG in a Kras-driven, p53-null genetically engineered mouse model and in patient-derived xenografts of LADC. Finally, we performed therapeutic trials in genetically engineered and patient-derived mouse models of LADC with the FDA-approved SGLT2 inhibitor canagliflozin. RESULTS/ANTICIPATED RESULTS: We observed a switch in the modality of glucose transport during lung carcinogenesis: SGLT2 was highly expressed in pre-malignant lesions and well-differentiated LADC, whereas GLUT1 was upregulated in advanced, poorly differentiated lesions. This pattern was observed both in human samples and in murine models. This observation led us to hypothesize that early-stage LADCs are often negative on FDG PET because this imaging modality does not detect the activity of SGLT2, which is expressed in early lesions. Therefore, we performed PET imaging with the tracer Me4FDG, that measures SGLT2 activity, in our mouse model, and observed that Me4FDG accumulated in small nodules that were negative with FDG. We confirmed the functionality of SGLT2 in human LADC by Me4FDG PET in patient-derived xenografts. To investigate the role of SGLT2-mediated glucose uptake in the early stages of LADC development, we treated both genetically engineered mice and patient-derived xenografts with FDA-approved SGLT2 inhibitors, showing that SGLT2 inhibition effectively reduced LADC growth and prolonged survival in mouse models. In addition, Me4FDG uptake predicted response to SGLT2 inhibition. DISCUSSION/SIGNIFICANCE OF IMPACT: Our results show that sodium-dependent glucose transport is a critical metabolic supply strategy in the early stages of lung adenocarcinoma development, and that Me4FDG is a novel biomarker of early LADC and of SGLT-dependent tumor growth. The discovery of SGLT2 in LADC highlighted the need for a re-interpretation of FDG-negative lung nodules, which might rely on SGLT2 for glucose uptake, and therefore may be detected by the new tracer Me4FDG. We anticipate our findings will lead to clinical studies evaluating Me4FDG as a diagnostic tracer for solitary lung nodules and early LADC, and as a biomarker for the selection of patients eligible for treatment with SGLT2 inhibitors.

